# Imaging of the Head and Neck following Radiation Treatment

**DOI:** 10.4061/2011/607820

**Published:** 2011-05-11

**Authors:** J. Matthew Debnam

**Affiliations:** Section of Neuroradiology, Department of Radiology, The University of Texas MD Anderson Cancer Center, Houston, Texas 77030, USA

## Abstract

Squamous cell carcinoma of the head and neck occurs in approximately 40,000 patients annually in the United States and is often treated with radiation therapy. Radiological studies are obtained following treatment for head and neck malignancies to assess for recurrent tumor, posttreatment changes, and associated complications. Radiation treatment creates a difficult clinical picture for oncologists, head and neck surgeons, neuroradiologists, and neuropathologists. As post-treatment imaging studies are often discussed at radiology/pathology working conferences, knowledge of the imaging appearance of radiation-associated changes in the head and neck and the terminology used by neuroradiologists may not only aid in interpretation of the pathologic specimen, but also assist in communications with neuroradiologists and referring clinicians.

## 1. Introduction

Squamous cell carcinoma of the head and neck is diagnosed annually in approximately 40,000 patients in the United States [1]. Radiation therapy is one component of the treatment for this disease. This treatment leads to endothelial damage and fibrosis, causing impairment of vascular and lymphatic flow [2], producing hypoxic, hypocellular, and hypovascular tissue. This tissue is unable to maintain normal tissue turnover [3, 4] resulting in tissue necrosis, infection, and ulceration [4–6]. Imaging of the head and neck in patients treated for malignancy is routinely performed to evaluate for recurrent tumor and treatment complications and is complementary to the physical examination. CT examinations can evaluate the underlying soft tissues and bony structures, which cannot be visualized on physical examination. 

There are a variety of complications following radiation treatment to the neck and it is important for neuropathologists involved with head and neck cancer to be aware of these complications. This paper reviews treatment-related changes including osteoradionecrosis of the mandible, hyoid bone, and skull base, discusses the imaging appearance of soft tissue ulceration and fistulous tract formation, as well as intracranial radiation injury and radiation-associated lesions. Knowledge or the imaging appearance of radiation-associated changes in the head and neck and the terminology used by neuroradiologists may aid in interpretation of the pathologic specimen and will assist in communications with neuroradiologists, head and neck surgeons, and referring clinicians.

## 2. Mucosal Irritation and Edema

**Figure 1:**
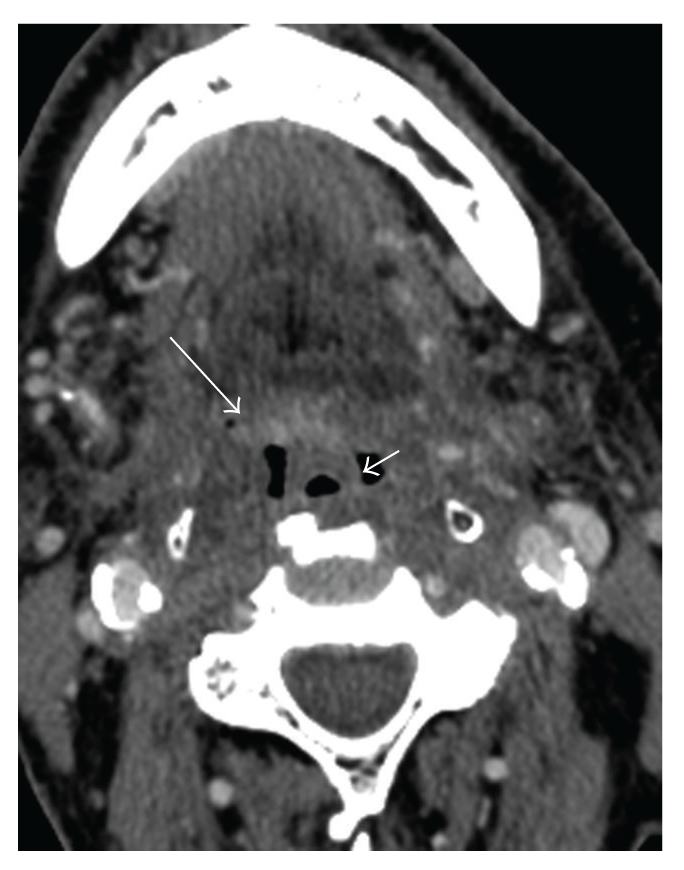
Postradiation changes of the oropharynx: Axial postcontrast CT demonstrates mucositis of the oropharynx characterized by enhancement (large arrow), and edema/swelling, or the epiglottis (small arrow).

Within the first two weeks of treatment, mucosal irritation and edema may occur. In the pharynx and larynx, mucositis and submucosal edema result in prominent mucosal contrast enhancement with thickening of the epiglottis and aryepiglottic folds (Figure 1). Fibrosis and atrophy develop over many more months and do not normalize even years after treatment [7]. Necrosis of the pharynx and larynx peaks in the first 12 months after treatment, but has been reported to occur more than 10 years after radiation therapy [8].

## 3. Osteoradionecrosis

Osteoradionecrosis, a known complication of radiation therapy for head and neck malignancies [9–11], involves the destruction of bony structures. The breakdown of collagen and cellular death overcomes the ability of the affected tissue to replicate and leads to failure of healing [3, 11]. This complication is frequently accompanied by infection, particularly when it occurs in the mandible. Synchronous or metachronous lesions can also occur in cancer patients, so it is important to determine that a lesion is within the radiation field before considering the diagnosis of osteoradionecrosis [12]. 

Risks for osteoradionecrosis related to the radiation therapy include total radiation dose, photon energy, brachytherapy, field size, fractionation [13]. Osteoradionecrosis is unlikely to occur if the radiation dose is below 60 Gy, delivered by standard fractions [14], but has a higher likelihood of occurrence if the dose is higher than 65–75 Gy [13]. Other risk factors for the development of osteoradionecrosis include periodontitis, poor oral hygiene, alcohol and tobacco use [15], dental extractions, tumor size, location and stage, proximity of tumor to bone, and preirradiation bone surgery [13]. Reuther et al. [16] studied 830 head and neck tumor patients evaluated during a 30-year period and suggested that tumor stage, infiltration of adjacent bone, and tooth extractions are the most important predisposing factors for osteoradionecrosis.

A study by Curi and Lauria [17] demonstrates that oral cancers showed the highest incidence of osteoradionecrosis. Seventy-eight percent of occurrences involved the tongue, retromolar trigone, and floor of mouth. This may be related to involvement of the mandibular bone in the radiation fields and the aggressive, often radical surgical approach necessary for tumor resection of these lesions. 

The clinical presentation of osteoradionecrosis includes pain, drainage, and fistula formation between the mucosa or skin, and related to bone in the radiation field. Other symptoms include otalgia, pain localized to the face, jaw, or throat. Long-term complication includes dry mouth, loss of taste, progressive periodontal attachment loss, dental caries, microvascular alternation, soft tissue necrosis, less commonly osteoradionecrosis, and limitation of mouth opening [13].

### 3.1. Mandible

Mandibular osteoradionecrosis (ORN) is a serious complication of radiation therapy for neoplasms of the oral cavity, oropharynx, nasopharynx, and parotid gland, with a varying reported incidence of 5% to 22% [9, 11, 18, 19]. The higher incidence of mandibular involvement has been attributed to its lower blood supply compared with the maxilla and the compact bone structure [11, 20].

**Figure 2:**
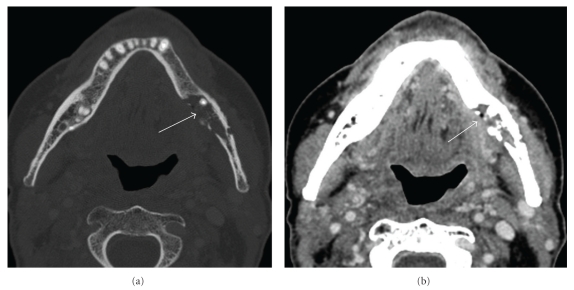
Mandibular osteoradionecrosis: (a) Axial contrast-enhanced CT of the mandible (bone window) shows destruction of the mandible, including along the lingual cortex and loss of the normal trabecular pattern (arrow). (b) Axial contrast-enhanced CT (soft tissue window) shows no evidence of an enhancing soft tissue mass. Linear enhancement (arrow) represents mucositis.

The clinical diagnosis of mandibular osteoradionecrosis is based on symptoms and signs of ulceration or necrosis of the overlying mucous membrane with exposure of necrotic bone [21]. Since a soft-tissue abnormality may be misinterpreted as tumor recurrence, correlation should be made with the typical osseous findings of mandibular osteoradionecrosis on CT scans. These include cortical disruption, disorganization of trabeculation, and osseous fragmentation [22] (Figure 2). Chong et al. [23] demonstrated that enhancement can occur in the soft tissues adjacent to osteoradionecrosis; however, identifying the aforementioned signs of osteoradionecrosis can lead to the correct diagnosis. Associated with osteoradionecrosis of the mandible may also be diffuse enhancement of the adjacent masseter and pterygoid muscles, and this should not be confused with tumor [23].

### 3.2. Hyoid  Bone

**Figure 3:**
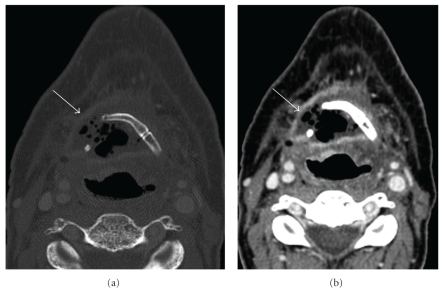
Hyoid bone osteoradionecrosis: (a) Axial contrast enhanced CT of the mandible bone (bone window) shows destruction of the right hyoid bone with soft tissue air (arrow). (b) Axial contrast-enhanced CT (soft tissue window) shows soft tissue ulceration without evidence of an enhancing soft tissue mass (arrow).

The hyoid bone is located inferior to the oral cavity and oropharynx and above the thyroid cartilage. The musculature of the floor of the mouth and the tongue are attached to the hyoid bone, providing assistance in tongue movement and swallowing. Tumor adjacent to the hyoid bone before radiation therapy is a factor that should be considered as putting the hyoid at risk [24] for osteoradionecrosis. Findings of hyoid osteoradionecrosis include fragmentation, cortical disruption, intraosseous or peri-hyoid air, often adjacent to a tongue-base ulceration. The absence of obvious enhancing soft-tissue tumor should suggest hyoid ORN in the radiated patient [24] (Figure 3).

### 3.3. Skull Base

**Figure 4:**
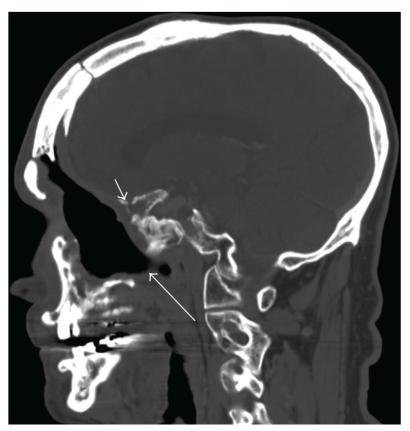
Radiation necrosis of the skull base with fistulous tract: Sagittal contrast-enhanced CT of the mandible bone (bone window) shows irregularity of the skull base (small arrow). In addition, there is a fistulous tract extending towards the oral cavity (large arrow).

Osteoradionecrosis of the skull base may be suggested by destruction of the bone and may be extensive and symmetric or localized. The most common locations are the sphenoid bone, followed by the clivus, internal carotid canal, and temporal bone. Destruction of bone is present with sequestra present within or surrounding necrotic bone and small collections of air within the soft tissue adjacent to the necrotic bone [25] (Figure 4). Huang et al. [25] reported that the pathologic evaluation is very important because sometimes it is difficult to differentiate tumor recurrence from ORN or the two pathologic changes existed at the same time during the operation.

## 4. Chondronecrosis

### 4.1. Larynx

**Figure 5:**
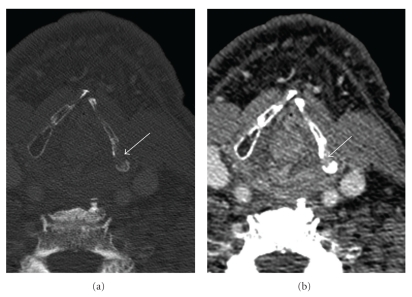
Chondronecrosis of the thyroid cartilage: (a) Axial contrast-enhanced CT of the thyroid cartilage (bone window) shows destruction of the left side of the cartilage (arrow). (b) Axial contrast-enhanced CT (soft tissue window) shows no evidence of an adjacent enhancing soft tissue mass (arrow).

The larynx includes the thyroid, arytenoid, and cricoids cartilages and is involved in speech and swallowing. Computed tomography can have a role in the evaluation of patients showing signs of laryngeal edema and/or necrosis after radiation. The diagnosis of chondronecrosis of the larynx can be strongly suggested in cases of sloughing of the arytenoid cartilage, fragmentation and collapse of the thyroid cartilage, and the presence of gas bubbles around the cartilage [12] (Figure 5). On the other hand, if CT scans show asymmetric laryngeal tissues in a symptomatic patient, these findings may be used to target the biopsy into the most suspect area radiologically, which may be an enhancing mass, increasing the specificity of biopsy findings [12].

## 5. Soft Tissue Injury

### 5.1. Ulceration

**Figure 6:**
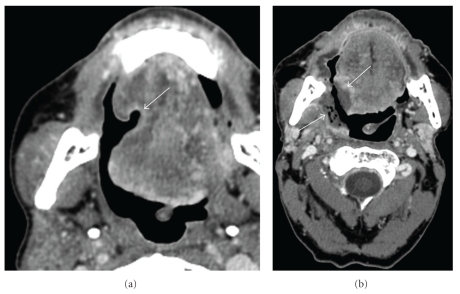
Benign and malignant soft tissue ulceration: (a) Axial contrast-enhanced CT of the oral cavity (soft tissue window) shows a benign ulceration (arrow) without an associated soft tissue mass. (b) Axial contrast-enhanced CT (soft tissue window) shows recurrent tumor characterized by irregular enhancement along the floor of the ulceration (arrows).

Ulceration is defined as a defect, or excavation, of the surface of a tissue or organ, which is produced by the sloughing of inflammatory necrotic tissue [26]. As most necrosis and many recurrences occur within 2 years following radiation therapy [26] and the risk of injury related to radiation necrosis is greatest during the first 6–12 months after radiation therapy [21, 27], time of onset of the ulceration is usually not helpful in distinguishing between radiation injury and recurrent tumor. Debnam et al. [28] studied the imaging findings of 20 patients with radiation-associated soft tissue ulcerations. They found that ulcerations without adjacent enhancement failed to demonstrate evidence of recurrent tumor, either with biopsy or on follow-up imaging. This finding suggests that an ulceration without adjacent enhancement is likely benign (Figure 6(a)). When ulcerations demonstrate adjacent enhancement, careful observation is required, if biopsy is not performed, as 4 of 8 ulcerations with adjacent enhancement demonstrated recurrent tumor (Figure 6(b)), while the other 4 ulcerations were free of tumor with biopsy or on follow-up imaging.

### 5.2. Fistula

**Figure 7:**
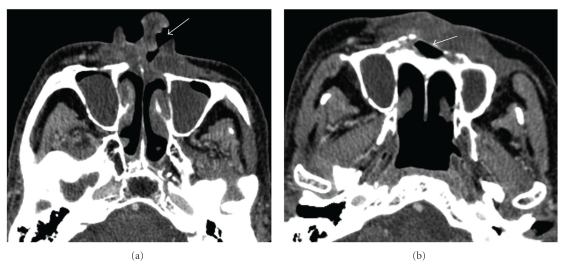
Orocutaneous fistula: (a, b) Axial contrast-enhanced CT of the oral cavity (soft tissue window) shows an orocutaneous fistula involving the left nasal ala (arrow) and extending through the maxilla (arrow) towards the oral cavity.

A fistulous tract is an abnormal pathway between an internal cavity or organ and the surface of the body. These may be caused by infection, tumor, or radiation. Orocutaneous fistulas (Figure 7) are not common, but intraoral sinus tracts due to dental infections are common [29]. An orocutaneous fistula leads to aesthetic problems due to the continual leakage of saliva from the oral cavity to the face. With the presence of an adjacent soft tissue mass, coexisting tumor cannot be excluded [30].

### 5.3. Thyroglossal Duct Cyst

**Figure 8:**
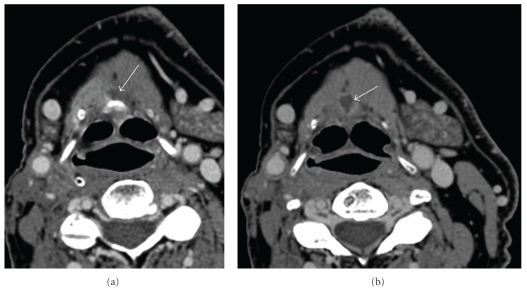
Enlarging thyroglossal duct cyst: (a, b) Axial contrast-enhanced CT at the level of the hyoid bone (soft tissue window) demonstrates a thyroglossal duct cyst (arrow) which has enlarged following radiation therapy and is containing more fluid centrally (arrow).

Seventy percent of congenital neck masses are thyroglossal duct cysts [31]. These can occur anywhere along the course of the thyroglossal duct, usually at the level of the hyoid bone. Enlargement of pre-existing thyroglossal duct cysts following radiation therapy has been reported by Singh et al. [32]. They noted that the enlarged thyroglossal duct cyst became more fluidlike (Figure 8) and should not be misinterpreted as a tumor. When sonographyguided biopsy was performed, microscopic evaluation revealed proteinaceous fluid and histiocytes, and a diagnosis of a benign cyst was made. The enlargement is believed to have an inflammatory cause and shrinkage or stability of the cyst suggests resolution of the inflammation.

## 6. Temporal Lobe Necrosis

**Figure 9:**
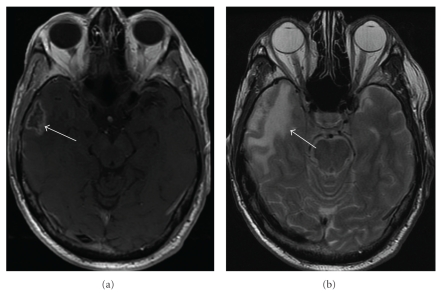
Temporal lobe radiation necrosis: (a) Axial contrast-enhanced MR examination of the brain demonstrates a peripherally enhancing focus in the right temporal lobe. (b) Axial fast spin echo T2 sequence shows edema around the focus of radiation necrosis characterized by signal hyperintensity (arrow).

Radiation necrosis to the temporal lobes of the brain can occur following radiation treatment of head and neck tumors, notably for lesions of the nasopharynx. The incidence has been reported to be 3% [33]. The earliest sign of temporal lobe necrosis is cerebral edema, which can be extensive [34]. Disparity between clinical and radiologic findings is noteworthy and highly suggestive of temporal lobe necrosis, and enhancing lesions can be located in the gray or white matter [34]. Together with an appropriate history, a presumptive diagnosis can be made, and pathologic proof in most cases is not required [35]. When treated early with corticosteroids, patients can make a complete or near complete recovery with only residual cerebral atrophy (Figure 9) [34].

## 7. Abscess

**Figure 10:**
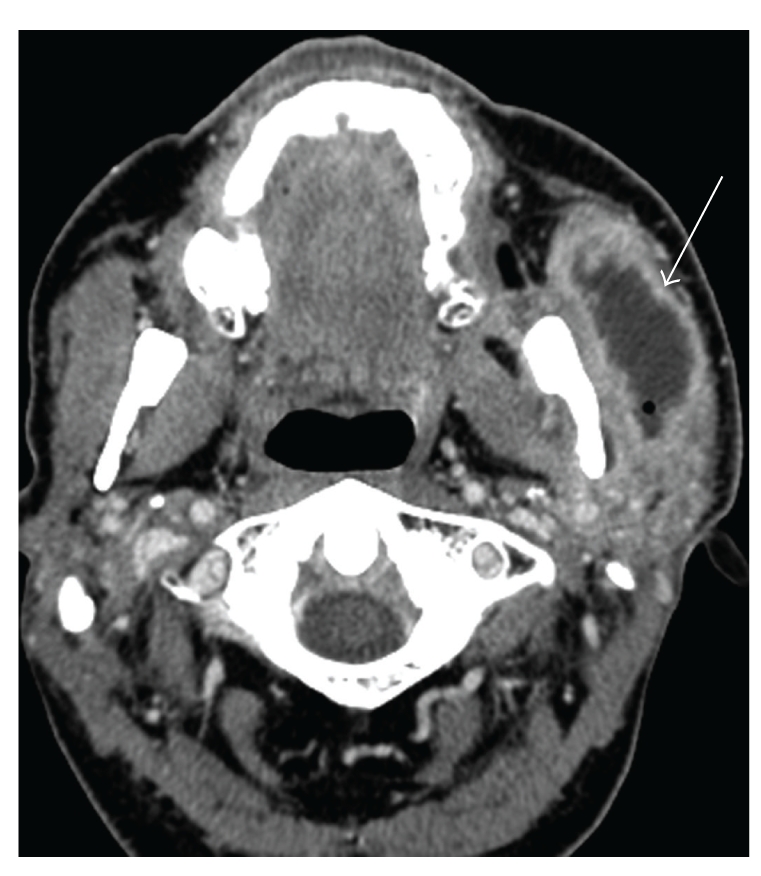
Left facial abscess: (a) Axial contrast-enhanced CT (soft tissue window) shows an abscess (arrow) of the left face. This is characterized by a peripherally enhancing fluid collection with a small focus of air and adjacent soft tissue swelling.

Abscess formation after radiation may be related to surgery or be odontogenic in origin. Abscesses will present as a rim enhancing fluid collection with a surrounding edema, characterized by soft tissue swelling and reticulation (Figure 10), and may be associated with osteomyelitis of adjacent bony structures [36].

## 8. Radiation-Associated Neoplasm

**Figure 11:**
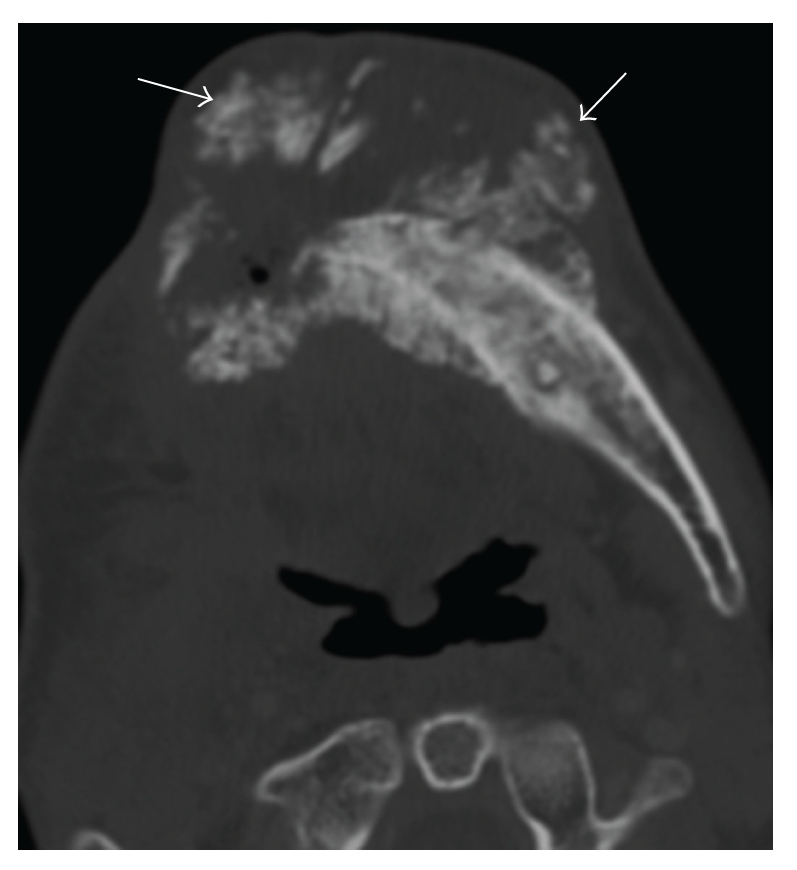
Radiation-associated osteosarcoma of the mandible: (a) Axial contrast-enhanced CT of the mandible (bone window) shows an osteoid matrix (arrow) within the tumor.

Sarcomas are a known complication [37, 38] of radiation therapy. These lesions arise in 0.035–0.2% of all irradiated patients [38] and represent less than 5% of all sarcomas [37]. A total dose of 55 Gy or above has been reported to increase the incidence of radiation-associated sarcomas [38]. These sarcomas may present as an enhancing soft tissue, defined mass and/or bone destruction (Figure 11).

## 9. Conclusion

Interpretation of the posttreatment neck can be difficult, with the appearances of complications sometimes mimicking recurrent tumor. A basic understanding of the findings commonly seen after radiation therapy may aid the neuropathologist in interpreting pathologic specimens. Neuropathologists will be aided by familiarity with the imaging appearances of the posttreatment neck, including changes to bone and soft tissue structures and features differentiating expected complications from recurrent tumor.
